# Associations of sedentary behavior and physical activity with psychological distress: a cross-sectional study from Singapore

**DOI:** 10.1186/1471-2458-13-885

**Published:** 2013-09-24

**Authors:** Robert A Sloan, Susumu S Sawada, Daniel Girdano, Yi Tong Liu, Stuart JH Biddle, Steven N Blair

**Affiliations:** 1 Health Promotion Board, Physical Activity Centre of Excellence, Singapore, Singapore; 2 Department of Health Promotion and Exercise, National Institute of Health and Nutrition, Tokyo, Japan; 3 Health Sciences Public Health, Walden University, Minneapolis, USA; 4 School of Sport, Exercise & Health Sciences, Loughborough University, Leicestershire, UK; 5 University of South Carolina, Departments of Exercise Science and Epidemiology /Biostatistics, Columbia, USA

**Keywords:** Sedentary behavior, Volume, Physical activity, Psychological distress, Singapore, Cross-sectional

## Abstract

**Background:**

Emerging evidence suggests the adverse association between sedentary behaviour (SB) with physical and mental health, but few studies have investigated the relationship between volume of physical activity and psychological distress. The present study examined the independent and interactive associations of daily SB and weekly level of moderate to vigorous physical activity (MVPA) with psychological distress in a multi-ethnic Asian population.

**Methods:**

De-identified data of 4,337 adults (18–79 years old) on sedentary behaviors, physical activity patterns, psychological distresses, and other relevant variables were obtained from the Singapore Ministry of Health’s 2010 National Health Survey. Psychological distress was assessed using General Health Questionnaire-12 (GHQ-12), whereas total daily SB and total weekly volume (MET/minutes) of MVPA were estimated using the Global Physical Activity Questionnaire version 2 (GPAQ v2). Multivariate logistic regression analyses were carried out to estimate the odds ratios (95% confidence intervals) of the independent and interactive relationships of SB and MVPA with prevalence of psychological distress.

**Results:**

The category of high SB was positively associated with increased odds (OR = 1.29, 1.04-1.59) for psychological distress, whereas the category of active was inversely associated with lower odds (OR = 0.73, 0.62-0.86) for psychological distress. Multivariate analyses for psychological distress by combined daily SB and weekly MVPA levels showed inverse associations between middle SB and active categories (OR = 0.58, 0.45 - 0.74) along with low SB and active categories (*OR* = 0.61, 0.47-0.80).

**Conclusions:**

The present population-based cross-sectional study indicated that in the multi-ethnic Asian society of Singapore, a high level of SB was independently associated with psychological distress and meeting the recommended guidelines for physical activity along with ≤ 5 h/day of SB was associated with the lowest odds of psychological distress.

## Background

The World Health Organization officials define good mental health status as, “a state of well-being in which every individual realizes his or her own potential, can cope with the normal stresses of life, can work productively and fruitfully, and is able to make a contribution to her or his community” [1]. Recent findings of the Singapore Mental Health Study reported that 12.0% of Singaporeans had at least one affective, anxiety, or alcohol use disorder [2] and according to the Singapore 2010 National Health Survey, 12.9% of citizens reported having psychological distress (based on a General Health Questionnaire-12 [GHQ-12] score > 3) [3]. Psychological distress has no formal definition or nosology [4] and has been described as an emotional disturbance that may impact on the social functioning and day-to-day living of individuals [5]. Psychological distress may act as an antecedent for affective disorders and is consistent with Hans Selye’s inverted U-shaped stress-distress model [4]. The apex of the curve represents the onset of distress and is the theorized point at which mental and physical health may begin to become compromised [6]. The expression and onset of psychological distress may vary across cultures [7,8] and at the population level it has been linked prospectively to all-cause mortality and Type II diabetes [9,10]. Because psychological distress may be part of a pathway to more severe mental and physical health problems, a need exists for public health practitioners and researchers to better understand the associations of health-related lifestyle behaviors with psychological distress.

Two lifestyle behaviors that deserve investigation regarding psychological distress are sedentary behavior (SB) and moderate to vigorous physical activity (MVPA). SB has been defined as any waking behavior characterized by an energy expenditure ≤ 1.5 metabolic equivalents (METs) while in a sitting or reclining posture [11]. MVPA can be characterized by an energy expenditure ≥ 3 METs while participating in leisure time, occupation/housework, or commuting activities [12,13]. Both SB and MVPA have been independently associated with an array of non-communicable diseases, decreased life expectancy, and poor mental health outcomes [12,14-20]. Significant evidence has shown a curvilinear risk reduction for a variety of health outcomes across varying durations of MVPA, summarising that some MVPA is better than none and more is better than some [12,21]. However, the collective evidence for the nature of the relationship of SB with health outcomes continues to emerge and remains unclear [15-19]. Based on the current SB evidence, researchers have hypothesized that accounting for MVPA, without consideration of a SB baseline, may have resulted in an incomplete and overstated assumption of health benefits [15-19] from which the term “active couch potato” has been coined [22]. The relationship of SB and MVPA with psychological distress remains vague.

Since 2010, three studies have been conducted investigating various domains of SB and physical activity with psychological distress [23-25]. A nationally representative study conducted in Scotland [23] found that recreational screen SB did not increase the odds of psychological distress (based on a GHQ-12 score of ≥ 4) per se, but was associated with higher GHQ-12 scores [26]. A similar relationship was found in a stratified analysis, whereby high recreational screen SB consistently demonstrated higher GHQ-12 scores across all levels of MVPA [24]. Researchers conducted a study based on working adults in the United Kingdom and reported an association of higher GHQ-12 scores in women who had greater than 7 hours/day of non-occupational SB. Investigators in Australia found that greater than 6 hours/day of occupational SB was independently associated with the higher odds of psychological distress [25].

Relatedly, Teychenne, Ball, and Salmon conducted a systematic review on the relationship of SB and depression [20]. While the quality and type of SB measures were mixed, the researchers surmised that the literature suggested a positive association between high SB and risk of depression. The researchers recommended that future studies needed to focus on the interrelationship between SB and MVPA with depression [20]. Recently, Falkner echoed this point by stating that researchers need to test for associations between SB and mental health outcomes while accounting for levels of MVPA [27]. To this point, overwhelming evidence exists that total volume of activity is more closely related to the full array health outcomes than to any particular component [12,21]. Therefore, to test the premise, we investigated both the independent and interactive associations of daily SB and weekly MVPA with psychological distress in a nationally representative sample of Singaporean adults who were 18–79 years old.

## Methods

### Data source

De-identified data were obtained from the Singapore Ministry of Health’s 2010 National Health Survey [3]. The survey is conducted every six years and is a nationally representative sample of the overall health status of non-institutionalized physically able Singaporean residents 18 to 79 years of age [3]. The Institutional Review Board of the Health Promotion Board approved the National Health Survey data for this observational study as exempt because the data were de-identified.

### Participants

Determining the sample consisted of a two-step modified stratified design. In step 1, a sample of 47,500 households was selected from the National Database on Dwellings [28] to match the national dwelling type distribution. Once matched, a random sample of 17,000 household addresses was selected. To collect an accurate count of the number of adults per household dwelling, each respective housing unit was visited. Step 2 consisted of a disproportionate stratified (age and ethnic group) random selection of adults as identified in step 1, according to Kish tables, 7,695 citizens located near six regional polyclinic testing centers were selected randomly for interviews. The Malay and East Indian ethnic groups were oversampled to ensure that prevalence estimates for these minority groups were reliable. To mitigate the bias of health literacy, different languages (English, Chinese, Malay, and Tamil) were used during the National Health Survey interviews, which included the embedded GHQ-12. The overall response rate from the initial random selection of citizens for the National Health Survey was 57.7% with a total of sample size (*N*) of 4,337 citizens completing the survey. The representative multi-ethnic Asian population mix consisted of (a) 74.1% Chinese, (b) 13.4% Malay, (c) 9.2% East Indian, and (d) 3.3% others. Exclusion criteria for the purpose of this study consisted of participants identified with missing data fields germane to the data analysis (*N* = 35).

### Psychological distress

The GHQ-12 has been internationally established as a valid and reliable instrument to measure psychological distress in the general adult population [8]. The instrument consists of 12 questions in the areas of general happiness, concentration, decision-making, strain, problem solving, self-confidence, and self-worth over the last six weeks. Interpretation of the answers was based on the accepted bimodal 4-point response scale (0 = *not at all*, 0 = *no more than usual*, 1 = *rather more than us*ual, and 1 = *much more than usual*). Based on validation studies conducted in Singapore [7] and internationally by the World Health Organization [8], the best threshold for detecting psychological distress was determined to be a GHQ-12 score of ≥ 2 (Sensitivity = 83.5% and Specificity = 75.1%).

### Sedentary behavior and moderate to vigorous physical activity

The Global Physical Activity Questionnaire version 2 (GPAQ v2) is a validated questionnaire [13] that was developed as a modification of the International Physical Activity Questionnaire [29] for use in multi-ethnic settings by the World Health Organization. The GPAQ v2 includes a single question used to estimate the daily hours/minutes spent participating in total daily SB, which includes sitting or reclining at work, at home, getting to and from places, or time with friends and time spent sitting at a desk, sitting with friends, travelling in car, bus, train, reading, playing cards or watching television, but does not include time spent sleeping [30-32]. This description of SB is consistent with the literature and provides a broad description of daily SB, which is not limited to a singular domain [16,22,31]. The GPAQ v2 was also used to estimate the total weekly volume (MET/minutes) of MVPA across three separate domains (work/home, during commuting, and during leisure time) performed for at least 10 minutes per session. The GPAQ v2 uses a complex set of algorithms to categorize weekly MVPA into three volumes of low (<600 MET/min), moderate (600–1,499 MET/min), and high (1,500 MET/min vigorous intensity or >3,000 MET/min MVPA). A detailed description of the calculation and categorization is at http://www.who.int/chp/steps/resources/GPAQ_Analysis_Guide.pdf[32]. Based on a recent global study [14], we further dichotomized and coded MVPA volume into two categories, *inactive* (less than a moderate volume) and *active* (moderate and high volume) for the multivariate analysis. The term active is considered to be synonymous with the World Health Organization’s health promoting recommendation for accumulating the equivalency of 150 minutes (2.5 h/wk) or more of moderate-intensity physical activity per week [12].

### Data analysis

Descriptive statistics were examined across daily SB tertiles. As recommended, cut points have not been established for SB, daily SB time was separated into tertiles of daily hours (h/day) to define the levels of low (2.5 h/day) middle (5 h/day) and high (10 h/day). First, we compared baseline characteristics of participants according to SB tertiles using one-way ANOVA for continuous variables and a Kruskal-Wallis test for categorical variables as appropriate (Table 1). Secondly, we used a multivariate logistic regression model to estimate the odds ratios (*OR*) and 95% confidence intervals (95% CI) of the independent relationships of SB and MVPA with prevalence of psychological distress presented along with *p* value trends (Table 2). Lastly, we used a multivariate logistic regression model to estimate the *OR* (95% CI) of the interactive relationship between SB and MVPA with prevalence of psychological distress (Table 3).

**Table 1:** Baseline characteristics according to sedentary behavior levels (tertiles)

**Variables**	**Total (*n *= 4,302)**	**Low (*n *= 1,211)**	**Middle (*n *= 1,687)**	**High (*n *= 1,404)**	***p *value**
Sedentary time (hrs/day)	5 (3–8)	2.5 (2–3)	5 (4–6)	10 (8–11)	
Age (years)	43 (33–54)	46 (37–56)	44 (34–55)	39 (29–49)	< 0.001^*^
Gender					< 0.001^*^
Male	2,084 (48.4)	502 (41.5)	818 (48.5)	764 (54.4)	
Female	2,218 (51.6)	709 (58.5)	869 (51.5)	640 (45.6)	
Body mass index (kg/m2)	25.4 ± 4.9	25.7 ± 4.9	25.6 ± 5.0	25.0 ± 4.7	0.001^†^
Weekly MVPA (1=low 2=moderate 3=high)	1.83 ± 0.74	2.01 ± 0.78	1.85 ± 0.73	1.65 ± 0.69	< 0.001^†^
Psychological distress					< 0.001^*^
< 2	3431 (79.8)	1002 (82.7)	1369 (81.1)	1060 (75.5)	
≥ 2	871 (20.2)	209 (17.3)	318 (18.9)	344 (24.5)	
Ethnic group					0.001^*^
Chinese	1,336 (31.1)	310 (25.6)	491 (29.1)	535 (38.1)	
Malay	1,285 (29.9)	427 (35.3)	522 (30.9)	336 (23.9)	
Indian	1,343 (31.2)	399 (32.9)	541 (32.1)	403 (28.7)	
Other	338 (7.9)	75 (6.2)	133 (7.9)	130 (9.3)	
Marital status					< 0.001^*^
Never married	879 (20.4)	148 (12.2)	334 (19.8)	387 (28.3)	
Married	3,091 (71.9)	950 (78.5)	1,298 (71.6)	933 (66.4)	
Separated	22 (0.5)	11 (0.9)	8 (0.5)	3 (0.2)	
Divorced	127 (3.0)	35 (2.9)	59 (3.5)	33 (2.4)	
Widowed	183 (4.3)	67 (5.5)	78 (4.6)	38 (2.7)	
Household income (Singapore dollar/month)					< 0.001^*^
<2000	1,095 (25.5)	375 (31.0)	495 (29.4)	225 (16.0)	
2000–3999	1,207 (28.1)	389 (32.1)	448 (26.6)	370 (26.4)	
4000–5999	741 (17.2)	173 (14.3)	284 (16.8)	284 (20.2)	
6000–9999	469 (10.9)	96 (7.9)	154 (9.1)	219 (15.6)	
>10000	297 (6.9)	31 (2.6)	103 (6.1)	163 (11.6)	
Refused	16 (0.4)	5 (0.4)	5 (0.3)	6 (0.4)	
Don’t know	477 (11.1)	142 (11.7)	198 (11.7)	137 (9.8)	
Chronic condition					< 0.001^*^
Yes	951 (22.1)	301 (24.9)	397 (23.5)	253 (18.0)	
Yes (not seeing doctor)	311 (7.2)	92 (7.6)	123 (7.3)	96 (6.8)	
No	3,040 (70.7)	818 (67.5)	1,167 (69.2)	1,055 (75.1)	
Smoking					0.530^*^
No	3,194 (74.2)	909 (75.1)	1,252 (74.2)	1,033 (73.6)	
Quit	356 (8.3)	92 (7.6)	130 (7.7)	134 (9.5)	
Occasionally	109 (2.5)	29 (2.4)	42 (2.5)	38 (2.7)	
Daily	643 (14.9)	181 (14.9)	263 (15.6)	199 (14.2)	
Binge drinking					< 0.001^*^
Non drinker	2,921 (67.9)	911 (75.2)	1,175 (69.7)	835 (59.5)	
Non binge drinker	1,078 (25.1)	231 (19.1)	387 (22.9)	460 (32.8)	
Binge drinker (≥5 drinks)	303 (7.0)	69 (5.7)	125 (7.4)	109 (7.8)	

Data represent median (IQR), mean ± SD, or number (percentages). *Kruskal Wallis, † ANOVA.

**Table 2:** Multivariate odds ratios for psychological distress according to sedentary behavior and MVPA levels

**Variables**	***N***	** Odds ratios**	** 95% CI**	**p for trend**
Daily sedentary behavior				0.013
Low	1,211	1.00 (Referent)*	―	
Middle	1,687	1.02	0.84 – 1.25	
High	1,404	1.29	1.04 – 1.59	
Weekly MVPA				<0.001
Inactive	1,611	1.00 (Referent)†	―	
Active	2,691	0.73	0.62 – 0.86	

*Adjusted for age, gender, BMI, ethnic group, chronic condition, marriage status, household income, smoking, binge drinking, and physical activity level.

†Adjusted for age, gender, BMI, ethnic group, chronic condition, marriage status, household income, smoking, binge drinking, and sedentary behavior.

**Table 3:** Multivariate odds ratios for psychological distress by combined daily sedentary behavior and total MVPA levels

**Variable**	**Participants**	**Odds ratios (95% CI)**
*High daily SB*		
Inactive	663	1.00 (Referent)
Active	741	0.82 (0.64 – 1.06)
*Middle daily SB*		
Inactive	589	0.93 (0.71 – 1.22)
Active	1098	0.58 (0.45 – 0.74)
*Low daily SB*		
Inactive	359	0.81 (0.58 – 1.12)
Active	852	0.61 (0.47 – 0.80)

Multivariate models were adjusted for confounding variables previously used in the literature [23,33] including age, gender, Asian body mass index (<18.5, 18.5-22.9, 23–27.4, ≥ 27.5), weekly MVPA (active, inactive), ethnic group (4 categories), chronic condition (3 categories), marriage status (5 categories), household income (7 categories), smoking (4 categories), and binge drinking (3 categories) categories [3]. All probability values of *p* < 0.05 were considered statistically significant. The Statistical Package for Social Sciences (version 17.0) was used for statistical analysis (SPSS, Inc., Chicago, Illinois, USA).

## Results

Table 1 shows baseline characteristics of the participants (*N* = 4,302) according to daily SB tertile levels. Those in the high SB tertile had the highest prevalence (24.5%) of psychological distress. Table 2 shows the independent odds ratios (*OR*, 95% CI) for the associations of daily SB and MVPA with psychological distress. After multivariate adjustment for potential confounding variables, the high SB category was independently associated with increased odds (*OR* = 1.29, 1.04-1.59) for psychological distress when compared to the referent low SB category. The magnitude and direction (*p* = 0.013) of the post hoc trend analysis indicated a collective positive association for higher daily SB with psychological distress. The active category was independently associated with decreased odds (*OR* = 0.73, 0.62-0.86) for psychological distress when compared to the referent inactive category. The magnitude and direction (*p* < 0.001) of the post hoc trend analysis indicated a collective inverse association for being active with lower psychological distress. Table 3 provides multivariate adjusted odds ratios for psychological distress by combined daily SB and weekly MVPA levels. The results of the analysis indicated interactive inverse associations for combined middle SB and the active categories (*OR* = 0.58, 0.45-0.74) and for combined low SB and active categories (*OR* = 0.61, 0.47-0.80).

## Discussion

The aim of this observational study was to investigate the associations of volume of daily SB and weekly MVPA with psychological distress in a nationally representative sample of the multi-ethnic Asian adult population of Singapore. Our findings indicated that persons reporting about 10 h/day of SB were 29% more likely to report psychological distress independent of being active and other confounders. Active individuals also had 27% lower odds of psychological distress independent of SB category and other confounders. The findings of the ad hoc magnitude and direction trends appear to suggest a possible independent positive association between higher amounts of SB and the odds of psychological distress. Conversely, the magnitude and direction trends for weekly MVPA appear to suggest a possible inverse association between being active and lower odds of psychological distress. The interactive association analysis was more indicative of volume and demonstrated that being active was not associated with lower odds off psychological distress for those individuals who reported 10 h/day of SB. The finding is line with the term “active couch” potato [22], whereby individuals can accumulate the recommended amount of health promoting MVPA but too much SB mitigates the health benefits. Additionally, the interactive analysis indicated that active individuals who accumulated 5 h/day or less of SB had ≈ 40% lower odds of psychological distress. In addition, specific levels of age, gender, household income, smoking, binge drinking, and chronic condition status showed independent associations. Notably, no associations were found with objectively measured Asian body mass index or ethnicity. To the best of our knowledge, this is the first study to examine both the independent and interactive associations of daily SB and weekly MVPA with psychological distress. Moreover, it is the first study in Asia to investigate SB with a health outcome.

Our findings are consistent with the limited literature on the various measures of SB and physical activity with psychological distress. Hamer, Stamatakis, and Mishra reported the cross-sectional relationship of recreational screen SB with psychological distress measured on the GHQ-12 in a nationally representative sample (*N* = 3,920) as part of the National Scottish Health Survey [23]. After full adjustment for confounding variables, the association between high recreational screen SB (> 4 h/day) and odds of psychological distress was not significant. Atkin, Adams, Bull, and Biddle [24] indicated that working adults in the UK (*N* = 2,707) reporting the highest level of daily recreational computer SB (>90 min/day) had the highest odds of psychological distress. In a study on the association of occupational SB in employees in Australia (*N* = 3,367), Kilpatrick, Sanderson, Blizzard, Teale, and Venn [25] found that employees with high (>6 h/day) amounts of occupational SB had significantly higher odds of moderate psychological distress. Only Hamer et al. reported on interactive associations [23] of daily recreational screen SB and weekly MVPA with GHQ-12 scores but not the odds of psychological distress per se. The investigators found that for the high category (>4 h/day) of recreational screen SB, higher GHQ-12 scores persisted across all weekly MVPA levels (<30 min/week, 30–120 min/week, ≥150 min/week) with the highest score prevalent in the combined category of low weekly MVPA and high recreational screen SB. In line with our findings, those in the active category (≥150 min/week) had the lowest psychological distress scores across all recreational screen SB categories. While none of the previously mentioned researchers investigated the odds associations of total volume of daily SB and weekly MVPA with psychological distress specifically, the trends for both occupational and non-occupational SB seemed to indicate that high amounts of SB are independently associated with risk of psychological distress. Some corroborative evidence exists for the interactive associations between SB and MVPA.

Although psychological distress and depression are two distinct mental health outcomes, they do share some similarities in their respective assessment scales [4,34]. Therefore it is useful to discuss some of the associations. Researchers have used accelerometry to objectively measure daily SB and found the combined categories of overweight/obese and high SB to be associated with higher odds of depressive symptoms [35,36]. The relevance of obesity as an effect modifier was further supported by researchers who investigated mental health status in older working and nonworking adults [37]. TV viewing in the obese working and the healthy weight nonworking populations was found to be associated with poorer mental health status. Notably, total SB was found to be associated with poorer mental health status in only the healthy weight nonworking population. Markedly, the relationship of SB and overweight/obese status with psychosocial distress was not apparent in our study or the related literature on SB and psychological distress [23-25].

The present study has several strengths. First, the study used nationally representative population data to examine the associations of daily SB and psychological distress in an Asian population, thus providing generalizability for the nation of Singapore and perhaps other similar Asian societies. The analysis conducted accounted for the covariates of gender (male and female), age (18–79 years old), Asian body mass index (BMI), and ethnic groups (Chinese, East Indian, and Malay), which are underrepresented in the literature [12]. Second, BMI was measured objectively; it has been found that self-reported BMI is underestimated for weight and overestimated for height [38]. Third, universally accepted measures of subjective daily SB and MVPA were used [14,30,31].

The primary limitation of this study is that it was cross-sectional design. Although the findings of the current study suggested a threshold effect, the nature of the study precludes causation. Therefore, prospective and experimental studies need to be conducted to better determine if SB operates as a predictor, indicator, or has a bidirectional relationship with psychological distress. For example, it is plausible to suggest that psychological distress could lead to greater SB and inactivity. Current study of SB and mental health outcomes is in infancy; therefore, more research is required. Secondly, participant bias cannot be eliminated based on the use of a self-report survey methodology; therefore, recall response bias may be evident. Because Singapore is a multiehtnic country that uses four distinct languages, interpretation bias cannot be ruled out. Additionally, we were unable to account for the variables of social interaction or nutrition status. Third, we were only able to assess total daily SB and not the construct of breaking up daily SB or domain of SB. Although our study demonstrated associations between volume of SB and MVPA, it is possible that specific SB and MVPA domains act in different ways on psychological distress.

Whether the mechanisms explaining the association between SB and MVPA with psychological distress are physiological, psychological, social, or all three is currently unclear; authors have suggested possible mechanisms of the link between poor metabolic health related to poorer mental health status [23] or social withdrawal [36]. The mechanism for the link between poor metabolic health and psychological distress deserves further investigation given that psychological distress [10] and SB [18] were each recently linked to Type II diabetes. Markedly, Asians have a higher risk for metabolic disorders at lower BMI levels than Caucasians [39] and are also suspected to have a genetic predisposition to lower fitness levels given similar MVPA levels as Caucasian counterparts, which was linked also to metabolic disorders [40].

An additional area needing further investigation is the relationship of social interaction SB and MVPA with psychological distress. Arredondo et al. [41] investigated the relationship of daily SB and MVPA with depression in Latinos residing in America and found that daily SB was related to the severity of depression and more so when social status was considered. Further research is needed using prospective and experimental designs to examine the potential causational relationship of SB and psychological distress. Beyond volume of SB, key questions exist regarding the relationship of breaking up SB throughout the day and its impact on psychological distress. Therefore, objective measures for measuring SB and MVPA should be used to understand this behavior better.

## Conclusions

Cross-sectional studies are frequently used to generalize the morbidity associated with a specific health risk along with the magnitude and distribution of a health problem in a population [42]. Therefore, the application of our findings adds and expands on the growing body evidence on the ill effects of too much SB [15,16,18,19] and may assist in the planning of health promotion services to increase physical activity and reduce SB. The results of our investigation confirmed the importance of distinguishing the independent and interactive associations of SB and MVPA on psychological distress. In conclusion, this population-based study indicated that in the multi-ethnic Asian society of Singapore, a high level of SB was independently associated with psychological distress and meeting the recommended guidelines for physical activity along with ≤ 5 h/day of SB was associated with the lowest odds of psychological distress.

## Competing interests

The authors declare that they have no competing interests.

## Authors’ contributions

RAS conceived of the study, performed the data mining, performed the statistical analysis, and contributed to the writing and editing of the manuscript. DG contributed to writing and editing of the manuscript. SSS contributed to the statistical analysis and editing of the manuscript. YTL contributed to the preparation of the manuscript. SJHB and SNB assisted in the writing and editing of the manuscript. All authors read and approved the final manuscript.

## Pre-publication history

The pre-publication history for this paper can be accessed here:

http://www.biomedcentral.com/1471-2458/13/885/prepub
